# Fluorinated Cell-Penetrating Peptide for Co-Delivering siHIF-1α and Sorafenib to Enhance In Vitro Anti-Tumor Efficacy

**DOI:** 10.3390/pharmaceutics15122789

**Published:** 2023-12-16

**Authors:** Yu Wan, Yuhan Yang, Qiuyue Lai, Wangxia Wang, Mingyu Wu, Shun Feng

**Affiliations:** Sichuan Engineering Research Center for Biomimetic Synthesis of Natural Drugs, School of Life Science and Engineering, Southwest Jiaotong University, Chengdu 610031, China; yuhanyang72@outlook.com (Y.Y.); qiuyue-lai@my.swjtu.edu.cn (Q.L.); wwx2021@my.swjtu.edu.cn (W.W.); wumy1050hx@swjtu.edu.cn (M.W.); fengshun@swjtu.edu.cn (S.F.)

**Keywords:** siRNA delivery, fluorination, peptide, hypoxia

## Abstract

Antiangiogenic therapy with sorafenib (SF) alone is ineffective in eradicating tumors, and its long-term application can exacerbate tumor hypoxia, which in turn restricts SF’s therapeutic efficacy. Here, a redox-responsive fluorinated peptide (DEN-TAT-PFC) consisting of dendritic poly-lysine, cell-penetrating peptide TAT, and perfluorocarbon was designed and synthesized to co-load siRNA-targeting hypoxia-inducible factors (siHIF-1α) and SF. The unique architecture of the peptide and fluorinated modifications enhanced the siRNA delivery efficiency, including increased siRNA binding, GSH-responsive release, cellular uptake, endosomal escape, and serum resistance. Simultaneously, the DEN-TAT-PFC/SF/siHIF-1α co-delivery system achieved efficient knockdown of HIF-1α at mRNA and protein levels, thus alleviating hypoxia and further substantially reducing VEGF expression. Additionally, the excellent oxygen-carrying ability of DEN-TAT-PFC may facilitate relief of the hypoxic microenvironment. As a result of these synergistic effects, DEN-TAT-PFC/SF/siHIF-1α exhibited considerable anti-tumor cell proliferation and anti-angiogenesis effects. Therefore, DEN-TAT-PFC can be a versatile platform for fabricating fluorine-containing drugs/siRNA complex nano-systems.

## 1. Introduction

Tumor progression causes the generation of a very complex pathological environment called a tumor microenvironment (TME), which directly promotes tumor growth. The rapid metabolism and proliferation of tumor cells require an increased blood supply to meet their demand for nutrients and oxygen. This leads to excessive oxygen consumption, thus creating a large disorganized and leaky tumor-associated vasculature and the formation of a hypoxic region [1]. This complex TME seriously interferes with the efficacy of common tumor therapies (e.g., causing chemotherapy resistance) [2], but also creates new ideas and possibilities for tumor treatment options [3,4].

For instance, antiangiogenic therapy relying on targeting vascular endothelial growth factor (VEGF) is a strategy for tumor treatment through the avoidance of new blood vessel formation [5]. However, a single antiangiogenic treatment strategy is ineffective in eliminating the tumor [6]. Moreover, long-term employment of sorafenib (SF), a multi-kinase inhibitor that suppresses tumor neo-angiogenesis by targeting VEGF, exacerbates tumor hypoxia [7]. And it has been found that clinically resistant hepatocellular carcinoma exhibited increased intratumoral hypoxia compared to pretreatment or sorafenib-sensitive hepatocellular carcinoma [8]. Alternatively, alleviating hypoxia appears to be a more promising strategy. Malignant tumor cells constantly adapt to hypoxic environments, a process mediated by a class of transcriptional activators called hypoxia-inducible factors (HIFs) [9]. One of the major isoforms, HIF-1α, is closely associated with tumorigenesis, progression, invasion, and metastasis, as up-regulated HIF-1α expression activates various downstream target genes [10], including activation of glucose transporter proteins GLUT1 and GLUT3 to promote glycolysis and VEGF to facilitate angiogenesis [11,12]. Hence, inhibition of HIF-1α has been considered an efficient approach to relieve hypoxia [13].

Given current challenges, we envision that integrating small interfering RNA (siRNA)-targeting HIF-1α (siHIF-1α), which silences the specific gene by RNA interfering, with SF can synergistically reconstruct TME by overcoming the hypoxia and angiogenesis to improve tumor treatment outcomes. However, due to their different properties, it requires a brilliant delivery system for co-delivering siRNA and small molecular drugs.

Poly-lysine and cell-penetrating peptides are common non-viral gene delivery vectors with a well-defined architecture, high density of surface charges, and good nucleic acid binding ability [14,15]. Nevertheless, cationic peptides have abundant positive charges that can cause severe cytotoxicity, and they are serum intolerant, easily forming large aggregates with proteins and other substances in the serum, resulting in reduced transfection efficiency [16]. The optimization of cationic carriers by architecture changes (e.g., branching, dendritic, or stellate), surface modifications (introduction of hydrophobic, and hydrophilic, fluorinated units, etc.), and the introduction of responsive linkers (disulfide bonds, ester bonds, etc.) can improve gene transfection efficiency and reduce cytotoxicity [17,18,19,20]. Especially, as in our previous study, fluorination modification of the peptide was a facile strategy to improve serum tolerance and promote cellular uptake and endosome escape, thereby improving transfection efficiency [21,22].

Coincidentally, it has been reported that fluorine-containing drugs can be loaded into fluorinated materials via interactions between fluorine atoms [23]. In this way, sorafenib-containing fluorine atoms can be loaded into the fluorinated peptide through F-F interactions. More intriguingly, perfluorocarbons (PFC) have unique oxygen storage and transport properties [24]. For example, Liu et al. applied erythrocyte membranes wrapped with PFC and poly(lactic-co-glycolic acid) to form nanoscale artificial red blood cells that could effectively transport oxygen to the tumor site after intravenous injection [25].

**Scheme 1:**
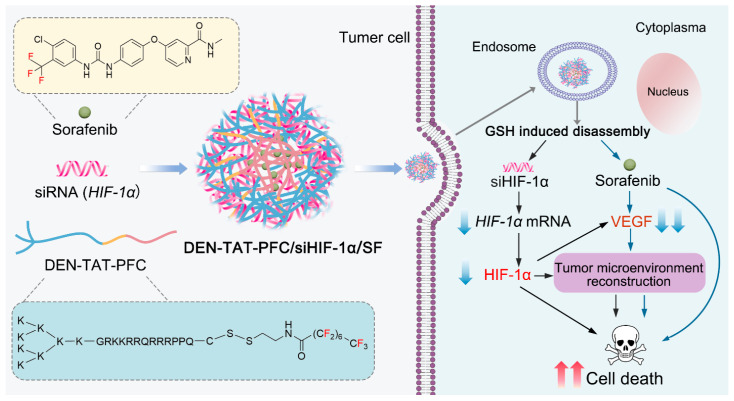
Schematic diagram of DEN-TAT-PFC for co-delivery of siHIF-1α and Sorafenib to enhance therapeutic efficacy by modulating tumor microenvironment.

Herein, a perfluorocarbon was linked to a dendritic poly-lysine (DEN) conjugated cell-penetrating peptide (TAT) through a disulfide bond to form a fluorinated peptide (DEN-TAT-PFC), which was employed for co-delivering SF and siHIF-1α, aiming at both vascular normalization and alleviating hypoxia for TME reconstruction, consequently combined gene therapy and chemotherapy synergistically enhances anti-tumor efficacy. PFC not only served as a component to enhance the efficiency of the peptide-based gene delivery vector and had a high affinity for the fluorinated drug SF, but also potentially played a role in this system by delivering oxygen and synergistically regulating the tumor hypoxic microenvironment (Scheme 1). The findings can provide references for constructing a co-delivery system for siRNA and fluorinated drugs and a targeting-hypoxia therapeutic strategy for tumor treatment.

## 2. Materials and Methods

### 2.1. Reagents and Materials

Bankpeptide Co., Ltd. (Hefei, China) custom-make the peptide (DEN-TAT) (sequence, K4K2KGRKKRRQRRRPPQC). 2-(2-Pyridyldithio)ethylamine hydrochloride (HY-101794-50) was purchased from MedChemExpress (Shanghai, China). Perfluorooctanoyl chloride and Cobalt chloride (CoCl_2_) were from Sigma-Aldrich (St. Louis, MO, USA). TrypLETM Express, Opti-MEM^®,^ and HEPES buffer were from Gibco (Waltham, MA, USA). Lipo8000™, DTT, Agarose, TBE buffer, LysoTracker Green, 100 × Hoechst 33342, Calcein AM cell activity assay kit, and Propidium iodide were obtained from Beyotime (Shanghai, China). Triton X-100 was purchased from Solarbio (Beijing, China). Matrigel^®^ matrix was from Corning (New York, NY, USA). Sorafenib was obtained from CSNpharm (Arlington Heights, IL, USA). Glutathione was purchased from Adamas-beta (Shanghai, China). DMOG was obtained from TCL. β-Actin antibody, HIF-1α antibody, and secondary antibody were purchased from Proteintech (Rosemont, IL, USA). siRNA-targeting VEGF (siVEGF) (anti-sense strand: 5′-GAUCUCAUCAGGGUACUCCdTdT-3′, sense strand: 5′-GGAGUACCCUGAUGAGAUCdTd-3′), siRNA-targeting HIF-1α (siHIF-1α) (sense strand: 5′-CGAUCAUGCAGCUAC UACAdT dT-3′; anti-sense strand: 5′-UGUAGUAGCUGCAUGAUCGdTdT-3′), Cyanine 5 labeled siRNA (Cy5-siRNA), and negative control scrambled siRNA (siNC) were all from Genepharma (Shanghai, China).

### 2.2. Synthesis of Fluorinated Peptide DEN-TAT-PFC

First, as in our previous study [21], N-(2-(2-pyridyldithio)ethyl)perfluorooctanamide (PFC) was produced. Then, the peptide DEN-TAT (5 mg, 1.76 μmol) was dissolved in distilled water containing 0.1% TFA, and PFC (1.2 mg, 2.06 μmol) was dissolved in acetonitrile containing 0.1% TFA, then the two solutions were mixed in equal volumes and reacted at room temperature (RT) for 4 h. The product was purified by semi-preparative reversed-phase high-performance liquid chromatography (RP-HPLC) (Figure S1) (YMC, Kyoto, Japan—Triart C18, S-5 µm, 10 × 250 mm, solvents: acetonitrile–water mixtures (0.1% (*v*/*v*) TFA), flow rate: 2.0 mL/min,) and the detailed solvent gradient was shown in Table S1. The separated DEN-TAT-PFC was freeze-dried with an ultra-low temperature freeze dryer for 48 h to obtain the desired product. Finally, the identity and purity of the obtained product were characterized by mass spectrometry ESI-MS and HPLC, respectively.

### 2.3. Gel Shift Assay

The siRNA compaction ability at various vectors/siRNA molar ratios (0, 2, 5, 10, 20, 50, and 100, 7.5 pmol siRNA) was evaluated by gel retardation assay. After dilution with 20 μL of the complexes in 20 mM HEPES buffer pH 7.2 (prepared with nuclease-free water), the vectors and siRNA were gently pipetted and incubated for 30 min at RT. The prepared 20 μL nanocomplexes were then gently mixed with 4 μL of 6 × DNA loading buffer, and the samples were loaded on a 1% agarose gel followed by electrophoresis at 100 V for 50 min. The gel was stained with ethidium bromide (EB) and observed by the iBright FL1500 Imaging System (Thermo Fisher Scientific, Waltham, MA, USA).

### 2.4. Serum Protection Assay

Scrambled siRNA-loaded DEN-TAT-PFC complexes prepared at a vector/siRNA molar ratio of 50 were selected for a serum protection assay. Complexes containing 7.5 pmol siRNA were incubated with 50% fetal bovine serum (FBS) for 0, 0.5, 2, 4, 6, and 24 h at 37 °C. A quick addition of 1 µL of 0.5 M EDTA was made to stop any nuclease activity. The siRNA remaining in the complexes was displaced with 1 µL of 50 μg/µL heparin to ensure the complete release of siRNA. Naked siRNA acted as a control and was treated in the same manner. As described above, the released siRNA was visualized by 1% agarose gel electrophoresis stained with EB.

### 2.5. siRNA Release Assay

To evaluate the siRNA release capacity, DEN-TAT-PFC/siRNA complexes formed with 7.5 pmol scrambled siRNA at a molar ratio of 50 were incubated at RT for 15 min with a 5 µL solution containing 0.5, 1, 2, 5, and 10 µg of heparin sodium. As a reference, 5 µL of nucleic acid-free pure water was added. In addition, the release profiles of siRNA from DEN-TAT-PFC/siRNA complexes (with 7.5 pmol scrambled siRNA at a molar ratio of 10) in reductive condition were further evaluated by gel electrophoresis assay after incubation with DTT (20 mM) for 4 h at 37 °C.

### 2.6. Assembly and Characterization of Vector/siRNA Complexes

DEN-TAT/siRNA and DEN-TAT-PFC/siRNA complexes were prepared by mixing siRNA and DEN-TAT or DEN-TAT-PFC gently at molar ratios of 50, 100, and 200 in HEPES buffer with 40 pmol siRNA to a final volume of 200 μL, and incubated for 30 min at RT before use. DEN-TAT-PFC/SF/siRNA complexes were prepared according to a DEN-TAT-PFC/siRNA molar ratio of 75, a siRNA content of 40 pmol, a final concentration of 25 μM of SF, and a final volume of 200 μL. First, the carrier and SF were gently pipetted and mixed well. Let it stand at RT for 30 min, then add siRNA and ultrapure water (nuclease-free, sterile) to the mixture, gently pipetted to mix evenly, and again let it stand for 30 min at RT to obtain DEN-TAT-PFC/SF/siRNA nanocomplexes. Next, the size distribution, as well as the zeta potential of complexes, was assessed by a dynamic light scattering (DLS) analyzer (Zetasizer, Malvern, UK).

### 2.7. Oxygen-Carrying Capacity Measurement

First, 3 mL of DEN-TAT and DEN-TAT-PFC solutions (2 mg/mL) was placed in a 5 mL centrifuge tube, and nitrogen was introduced into the solution to expel oxygen dissolved in the solution. The oxygen contents in these solutions were detected as a control. Next, oxygen was introduced into the solution until it was saturated with oxygen, and then ultrapure water, rapidly cooled after boiling, was added, and the change in dissolved oxygen contents in the solution was detected by a portable dissolved oxygen meter and monitored continuously for 10 min.

### 2.8. Transmission Electron Microscopy

The morphology of DEN-TAT-PFC/SF/siRNA complexes was observed by TEM (Jeol Ltd., Tokyo, Japan—Jem-f200). The sample solution was dropped on the TEM grid. After a few minutes, the excess solution was blotted away with filter paper. Then, 0.5% (*w*/*v*) of phosphotungstic acid was placed on the above grids. The grid was dried at RT for several minutes before observation.

### 2.9. Determination of Encapsulation Rate and Drug Load Efficiency of DEN-TAT-PFC/SF/siHIF-1α Nanocomplexes

HPLC was used to analyze the EE (%) and DLE (%) of nanocomplexes. The obtained nanocomplexes were centrifuged at 4 °C at 143,000× *g* for 15 min to remove free SF. The free SF concentration was determined using HPLC. Chromatographic conditions: C18 column (4.6 mm × 250 mm, 5 μm, Dikma, Beijing, China); Mobile phase: acetonitrile: water = 75:25 (*v/v*); Detection wavelength: 265 nm; Column temperature: 25 °C; Flow rate: 1.0 mL/min; Injection volume: 30 μL. The encapsulation efficiency (EE %) and drug load efficiency (DLE %) of nanocomplexes were calculated as follows:

$$\mathrm{EE}\;\left(\%\right)=\left(\frac{\mathrm{Total}\;\mathrm{amount}\;\mathrm{of}\;\mathrm{SF}-\mathrm{Amount}\;\mathrm{of}\;\mathrm{free}\;\mathrm{SF}}{\mathrm{Total}\;\mathrm{amount}\;\mathrm{of}\;\mathrm{SF}}\right)\times 100\%$$

$$\mathrm{DLE}\;\left(\%\right)=\left(\frac{\mathrm{Total}\;\mathrm{amount}\;\mathrm{of}\;\mathrm{SF}-\mathrm{Amount}\;\mathrm{of}\;\mathrm{free}\;\mathrm{SF}}{\mathrm{Total}\;\mathrm{weight}\;\mathrm{of}\;\mathrm{DEN}-\mathrm{TAT}-\mathrm{PFC}/\mathrm{SF}/\mathrm{siRNA}}\right)\times 100\%$$

### 2.10. In Vitro Release Profile of DEN-TAT-PFC/SF/siHIF-1α Nanoparticles

In vitro SF release from DEN-TAT-PFC/SF/siHIF-1α nanocomplexes was analyzed by dialysis. The prepared nanocomplexes were added to dialysis bags (molecular weight cut-off: 3500 Da) and then immersed in 11 mL PBS (pH 7.4) with or without 10 mM GSH containing 0.5% *w/v* Tween 80. Different sets of dialysis bags were placed in a 37 °C thermostatic shaker and shaken at 90 g. Then, 300 μL of dialysate was removed at preset time points of 0 h, 1 h, 4 h, 10 h, 24 h, 48 h, and 72 h, respectively, and replenished with equal amounts of the fresh-release medium. After the sampling was completed at each point, the solution in the dialysis bag was added to the same volume of methanol, the demulsification was carried out by ultrasound for 10 min, and 300 μL of the same sample was taken to calculate the total drug content. HPLC analysis was performed on samples from the above sampling points and demulsification samples. The conditions of HPLC analysis were the same as that in Section 2.9. The in vitro release curve of SF was plotted according to the sampling time point and the percentage of total drug content.

### 2.11. Hemolysis Assay

The hemolysis rate test was performed according to the following steps. First, 10 mL of fresh rat blood was taken into a vacuum blood collection tube coated with sodium heparin, mixed evenly by inverting slightly, centrifuged at 1000× *g* for 10 min after mixing, and then the supernatant was discarded. The above red blood cells (RBCs) were resuspended with PBS (about 10 times the volume of RBC) and centrifuged at 888 g for 10 min, washed 3 times until the supernatant was clear, and diluted with PBS to make a 2% RBC suspension. Then, 20 μL of 50 μM, 100 μM, and 200 μM peptides were added to 180 μL of 2% RBC suspension, which was mixed gently and incubated in a 37 °C water bath for 1 h, then centrifuged at 1065× *g* for 5 min. The optical density (OD) of the supernatant was read at 545 nm using enzyme labeling. The positive control (100% lysis) was a blood/Triton X-100 mixture, and the negative control (0% lysis) was a blood/PBS mixture. The hemolytic ratios of the samples were calculated as follows:

$$\mathrm{Hemolysis}\;\left(\%\right)=\left(\frac{\mathrm{Absorbance}\;\mathrm{of}\;\mathrm{sample}-\mathrm{Absorbance}\;\mathrm{of}\;\mathrm{negative}\;\mathrm{control}}{\mathrm{Absorbance}\;\mathrm{of}\;\mathrm{positive}\;\mathrm{control}-\mathrm{Absorbance}\;\mathrm{of}\;\mathrm{negative}\;\mathrm{control}}\right)\times 100\%$$

### 2.12. In Vitro Cytotoxicity

The cytotoxicity of vectors was evaluated against HepG2 and LO2 cells using MTT Assay. The cells were seeded in 96-well plates so that the cell density could reach around 60% after 24 h. Residual serum was washed away with sterile PBS, and 100 μL of Opti-MEM medium containing various formulations (different concentrations of vectors or different complexes) was added. After 4 h, 100 μL of 10% DMEM medium and 10 μL of FBS were added. Cells were continued to be cultured for 24 h or 48 h. Then, 10 μL of MTT solution (5 mg/mL) was added on a clean table protected from light and incubated in a cell incubator at 37 °C for 4 h, and blue-violet formazan crystals appeared at the bottom of the plate. The medium was carefully aspirated, then 150 μL of DMSO was added per well and incubated at 37 °C, 50 g for 10 min until the crystals were completely dissolved. The absorbance (A) of each well at 570 nm was measured with a microplate reader, and the cell viability was calculated as follows:

$$\mathrm{Cell}\;\mathrm{viability}\;\left(\%\right)=\left(\frac{\mathrm{Asample}-\mathrm{Ablank}}{\mathrm{Acontrol}-\mathrm{Ablank}}\right)\times 100\%$$

### 2.13. In Vitro Cell Uptake and Intracellular Tracking Study

The cells were seeded in 96-well plates to ensure the cell density could reach about 70–80% after 24 h. Complexes with various vectors/siRNA molar ratios were added to the cells in the Opti-MEM medium at the Cy5-siRNA concentration of 50 nM per well or in the 10% FBS medium at the Cy5-siRNA concentration of 100 nM per well. After incubation at 37 °C for 4 h, cells were washed three times with 200 μL FACS buffer (0.5 mg/mL heparin sodium, 2% (*v*/*v*) FBS in PBS) and once with PBS. Next, 25 µL TrypLETM was added to each sample and incubated for 5 min at 37 °C. Following that, 150 µL FACS Buffer was introduced to every well to obtain the cells, and then the cell uptake efficiency of Cy5-siRNA was assessed by flow cytometry (Accuri, Ann Arbor, MI, USA—C6, BD with FL4 detector (640/675 nm)).

The cell uptake was also evaluated by confocal laser scanning microscopy (CLSM). HepG2 cells were seeded into a confocal dish, and various Cy5-labeled siRNA-loaded nanocomplexes at the molar ratio of 100 (Cy5-siRNA 100 nM per well) were added. After incubation at 37 °C for 4 h, cells were washed with FACS buffer three times and once with PBS to remove the nanocomplexes that were not taken. The lysosomes and nucleus were stained with LysoTracker Green (200 nM) and Hoechst 33342 (1×), respectively, and then the cells were observed by CLSM.

### 2.14. In Vitro Gene Silencing

HepG2 cells were seeded in a 12-well (mRNA extraction) or 6-well (protein extraction) tissue culture plate and cultured overnight. Then, the complete medium was replaced by Opti-MEM medium or 10% FBS medium, followed by the addition of various complexes (with the siVEGF or siHIF-1α concentration of 100 nM per well at a molar ratio of 75 and SF concentration of 25 μM per well). Lipo8000^TM^ was used following the protocol as a control group. After incubation for 4 h, CoCl_2_ (100 μM) was added (HIF-1α mRNA and protein extraction) and further cultured for 48 h in serum-containing DMEM. HepG2 cells without treatment were used as a negative control. The expression of VEGF and HIF-1α were determined at mRNA (expression of VEGF and HIF-1α) and protein (expression of HIF-1α) levels by quantitative real-time PCR and Western blot assay as described below.

### 2.15. Quantitative Real-Time PCR

A Steady Pure Quick RNA Extraction Kit (Code No. AG21023) was used to isolate and purify total RNA from samples. Reverse transcription was carried out to create complementary DNAs (cDNAs) employing a reverse transcription kit (Code No. AG11728, Evo M-MLV RT Mix Kit with gDNA Clean), according to manufacturer’s instructions. Subsequently, qPCR experiments were conducted using the SYBR^®^ Green Premix Pro Taq HS qPCR Kit (Rox Plus) (Code No. AG11718-S; Accurate Biotechnology Co., Ltd, Hunan, China) on Real-Time PCR instrument (Thermo Fisher Scientific, Waltham, MA, USA—Step One Plus^TM^ Real-Time PCR System) as directed by manufacturer’s instructions. A separate real-time PCR using primers for detecting GAPDH (expression of VEGF) and β-actin (expression of HIF-1α) was used as a control. All PCR experiments were performed in triplicate, and the expression levels of genes were calculated against the GAPDH or β-actin. Specific primer sequences for VEGF, HIF-1α, GAPDH, and β-actin are listed in Table S2.

### 2.16. Western Blot Assay

Cells were washed once with cold PBS and then resuspended in RIPA lysis buffer (200 μL), freshly supplemented with complete protease inhibitor cocktail tablets and DMOG (1 mM). The lysates were sonicated until the lysate was clear and non-viscous. Total protein was separated on a 6% Bis-Tris-polyacrylamide gel and then transferred to PVDF membrane at 100 V. After incubation in 5% skimmed milk in 1 × TBST for 1 h, the membrane was incubated in 5% skimmed milk in 1 × TBST with a monoclonal antibody against HIF-1α (1:3000) overnight. The membrane was further incubated in 5% skimmed milk with goat anti-rabbit IgG (1:3000) for 1 h. Immunoreactive complexes were visualized using ECL reagents. β-actin (1:10,000) was used as a loading control.

### 2.17. In Vitro Angiogenesis Assay

A tube formation assay was employed to assess the effect of various formulations on human umbilical vein endothelial cells (HUVEC) vascularization. Liquefied Matrigel matrix was first placed into 48-well plates (150 µL per well), followed by 30 min incubation at 37 °C for solidifying. HUVECs were resuspended with the supernatants of HepG2 cells (condition medium) after transfection with different formulations (siRNA concentration in each well was 100 nM, the molar ratio of vectors to siRNA was 75), then the cells were added into the solidified Matrigel matrix, and incubated for a further 2~4 h. Images of tubule formation were observed by an inverted bright field microscope, and the total length (TL) was processed by Image J (2.3.0/1.53q) software. The rate of tubule structure formation was calculated using the following formula:

Tubule structure formation rate (%) = (TL_control_ − TL_sample_)/TL_control_ × 100%

### 2.18. Statistical Analysis

Data were expressed as mean ± standard deviation (SD) for at least three independent experiments. The differences in each group were statistically analyzed by one-way or two-way analysis of variance (ANOVA) by GraphPad Prism 9. When *p* < 0.05, the results were regarded as statistically significant (* *p* < 0.05, ** *p* < 0.01, *** *p* < 0.001, **** *p* < 0.0001).

## 3. Results and Discussion

### 3.1. Synthesis and Characterization of DEN-TAT-PFC

#### 3.1.1. Synthesis of DEN-TAT-PFC

**Figure 1:**
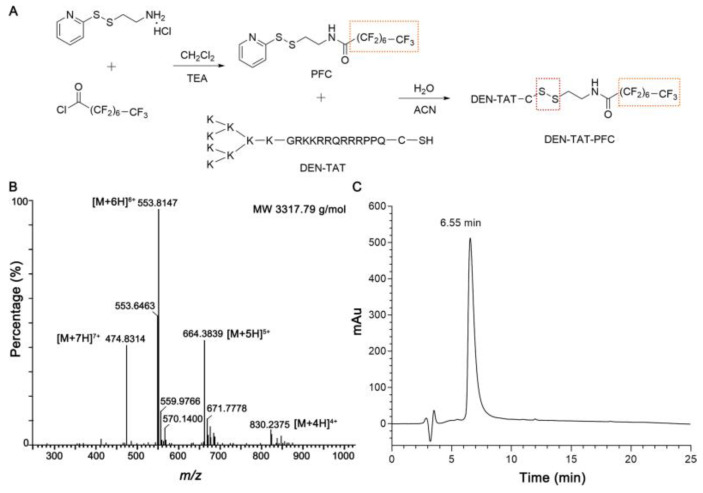
Synthesis of DEN-TAT-PFC: (**A**) Synthetic route of DEN-TAT-PFC. The identity (**B**) and purity (**C**) of the DEN-TAT-PFC were confirmed by LC-MS and HPLC (Dikma HPLC Column, C18, 5 μm, 4.6 × 250 mm; flow rate: 1 mL/min; detection wavelength: 214 nm; Solvent A:0.1% (*v*/*v*) TFA in water; Solvent B: 90% (*v*/*v*) acetonitrile, 0.1% (*v*/*v*) TFA in water; Gradient: 35% solvent B to 75% solvent B over 25 min)), respectively.

As in the previous study [21], using pyridyl disulfide reaction chemistry, the PFC was effectively conjugated to DEN-TAT while concurrently introducing the disulfide linkages (Figure 1A). The correct identity of the final product was confirmed by LC-MS (Figure 1B), and its purity was assessed via HPLC (Figure 1C; >95% pure). Additionally, the efficiency of PFC conjugation was about 75%.

#### 3.1.2. siRNA Binding and Release Capacity of DEN-TAT-PFC

**Figure 2:**
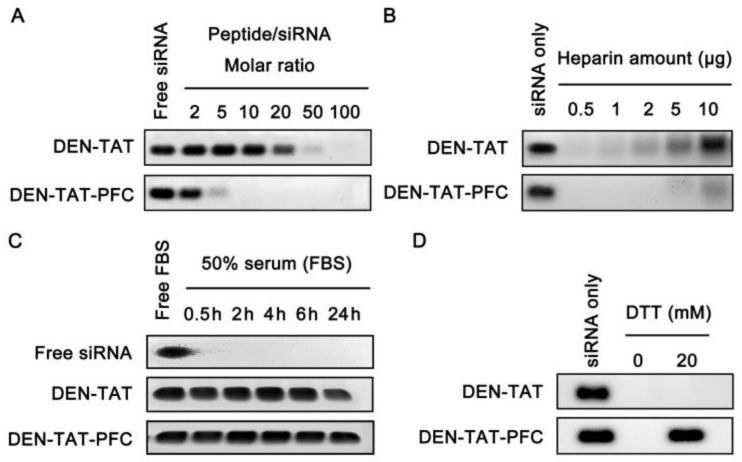
Characterization of vector/siRNA complexes: (**A**) Gel retardation assay results of DEN-TAT/siRNA complexes and DEN-TAT-PFC/siRNA complexes. (**B**) Comparing the release of siRNA from DEN-TAT/siRNA complexes and DEN-TAT-PFC/siRNA complexes following heparin incubation at varying concentrations. (**C**) The capacity of DEN-TAT and DEN-TAT-PFC to protect siRNA at various time points from treatment with 50% FBS. (**D**) siRNA release from DEN-TAT-PFC/siRNA complexes (molar ratio of 10) after treatment with 20 mM DTT.

siRNA binding and release capacity of DEN-TAT-PFC was investigated by a gel shift assay. Complete retardation of siRNA movement was observed at molar ratio 10 for DEN-TAT-PFC/siRNA complexes (Figure 2A). In comparison, DEN-TAT was unable to completely retard siRNA movement at the same ratio and even at a higher molar ratio of 50 (Figure 2A), suggesting that DEN-TAT-PFC was more efficient for siRNA complexation than its non-fluorinated analogue. A competitive heparin displacement experiment, in which complexes were treated with increasing levels of heparin as an electrostatic competition agent, was conducted to further assess the interaction between peptides and siRNA. The results (Figure 2B) demonstrated that a small amount of siRNA was released from DEN-TAT/siRNA complexes at heparin concentrations of 0.5 μg/20 μL, and the siRNA in DEN-TAT/siRNA complexes was fully competitively released upon the addition of heparin mass up to 10 μg/20 μL. In comparison, a tiny fraction of siRNA was released from DEN-TAT-PFC/siRNA complexes at 5 μg/20 μL assay, suggesting that the siRNA complexes with DEN-TAT-PFC were more stable than with DEN-TAT.

The stability of free siRNA and two-peptide/siRNA complexes against serum nucleases was assessed by incubating with 50% (*v*/*v*) serum (Figure 2C). It can be found that free siRNA was readily degraded in a high serum environment. In contrast, DEN-TAT and DEN-TAT-PFC could protect siRNA from degradation. Next, the redox-responsive release performance was investigated by incubation with 20 nM DTT. As shown in Figure 2D, adding DTT could not cause the release of siRNA from the DEN-TAT/siRNA complexes due to the absence of disulfide bonds in DEN-TAT. Nevertheless, the disulfide bond in DEN-TAT-PFC could break and allow the smooth release of siRNA in the presence of 20 mM DTT, because the removal of PFC reduced the binding affinity of the vector and siRNA.

#### 3.1.3. Characterization of DEN-TAT-PFC/siRNA Complexes

**Figure 3:**
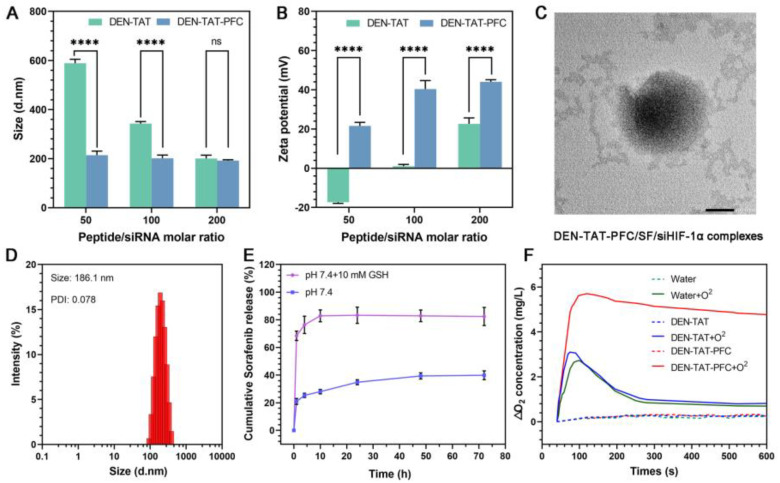
Characterization of DEN-TAT-PFC and its siRNA/drug Complexes: (**A**) The average hydrodynamic diameters and (**B**) ζ—potential of the DEN-TAT/siRNA complexes and DEN-TAT-PFC/siRNA complexes at various molar ratios. Results are displayed as mean ± SD (*n* = 3). (**C**) TEM images (scale bar 100 nm) and (**D**) the size distribution by the intensity of DEN-TAT-PFC/SF/siHIF-1α. (**E**) The accumulative release curves of Sor from DEN-TAT-PFC/SF/siHIF-1α in pH 7.4 PBS in the absence or presence of 10 mM GSH. (**F**) The oxygen loading capacities of DEN-TAT and DEN-TAT-PFC were estimated by recording the corresponding oxygen concentrations of various solutions using a portable dissolved oxygen meter. Data are expressed as mean ± SD (n = 3), ns represents insignificant, **** *p* ≤ 0.0001.

The particle size and zeta potential for vector/siRNA complexes were analyzed using DLS (Figure 3A,B). The size of the DEN-TAT/siRNA complexes decreased from approximately 600 nm to 200 nm with the increasing molar ratio from 50 to 200, while the size of the DEN-TAT-PFC/siRNA complexes changed slightly from about 214 nm to 191 nm. In addition, the zeta-potential of the DEN-TAT/siRNA complexes was −17 mV at a molar ratio of 50, approached 0 mV at a molar ratio of 100, indicating the complete condensation, and then reached 22 mV at a molar ratio of 200. On the contrary, the zeta-potential of the DEN-TAT-PFC/siRNA complexes was positive from a molar ratio of 50 to 200, reaching approximately 44 mV at a molar ratio of 200. These were in good agreement with the results of the above agarose gel electrophoresis experiments.

After clarifying the high siRNA binding ability of DEN-TAT-PFC, the co-delivery system (DEN-TAT-PFC/SF/siHIF-1α) was prepared and characterized. The encapsulation efficiency of SF was 97.46% ± 0.186%, and the drug-loading efficiency of SF was 20.48% ± 0.039%. The formed nanocomplexes exhibited a spherical-like structure (Figure 3C), a narrow size distribution with an average diameter of ~186 nm (Figure 3D), and positively charged surfaces (+28.4 mV) (Figure S1). Subsequently, the release behavior of SF from DEN-TAT-PFC/SF/siHIF-1α was investigated (Figure 3E). In the absence of GSH, the percentage of cumulative SF release only reached nearly 28.10% at 10 h and 39.85% at 72 h, with no tendency for further release. However, in the presence of 10 mM GSH, SF released rapidly, reaching approximately 68.43% at 1 h and 82.42% at 72 h, respectively, suggesting the GSH-triggered drug release property of this system.

A portable dissolved oxygen meter was performed to determine the oxygen-carrying capacity of the fluorinated carrier DEN-TAT-PFC (Figure 3F), and water was used as a negative control. The dissolved oxygen concentration of DEN-TAT-PFC after oxygen saturation treatment increased by 5.70 mg/L within 120 s, while that of the DEN-TAT solution only increased by 2.73 mg/L, which was not significantly different from that of the pure water solution. Moreover, the dissolved oxygen of DEN-TAT-PFC only decreased by 1.03 mg/L in the subsequent 480 s, while the dissolved oxygen of the DEN-TAT solution and water decreased super rapidly, indicating that DEN-TAT-PFC could be applied as an effective oxygen delivery vehicle and also could slowly release O_2_, which can be utilized to assist in alleviating the tumor hypoxic microenvironment.

### 3.2. Biosafety of DEN-TAT-PFC

**Figure 4:**
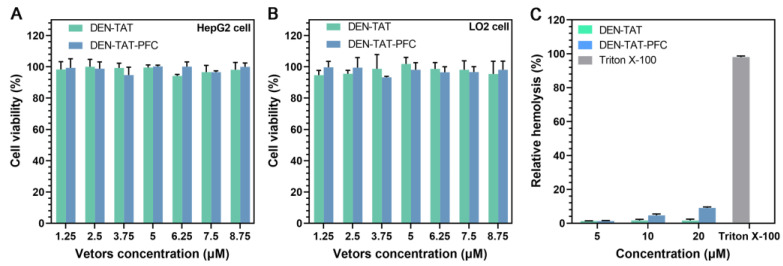
The biosafety of DEN-TAT-PFC. The viability of HepG2 cells (**A**) and LO2 cells (**B**) was determined after exposure to DEN-TAT and DEN-TAT-PFC with a concentration from 1.25 μM to 8.75 μM. (**C**) Hemolysis rate of DEN-TAT and DEN-TAT-PFC at different concentrations and the Triton X-100 was used as a positive control.

The cytotoxicity of delivery systems toward HepG2 and LO2 cells was assessed to evaluate biocompatibility. As shown in Figure 4A,B, DEN-TAT and DEN-TAT-PFC did not affect the viability of HepG2 and LO2 cells at concentrations ranging from 1.25 to 8.75 μM, and cell viabilities were maintained above 90%. The live-dead stain plots of HepG2 cytotoxicity allowed a more visual observation that both vectors revealed good biosafety at a high concentration of 8.75 μM (Figure S3A).

To further investigate the biosafety of the delivery systems, a hemolysis assay was carried out (Figure 4C and Figure S3B). Examining membrane leakage is an essential indicator of the cytotoxicity of cationic carriers. When the concentration of DEN-TAT-PFC was 20 μM, slight hemolysis was observed due to its higher membrane-disrupting activity, which was partially responsible for its higher cellular uptake and endosomal escape capacity, which will be discussed below. However, when the concentration of DEN-TAT-PFC was below 10 μM, the hemolysis rate was less than 5%, indicating that it was safe and non-hemolytic as biomaterials in this concentration range. Thus, DEN-TAT-PFC less than 10 μM was utilized in the following experiments.

### 3.3. In Vitro Gene Delivery Efficiency and Serum Resistance Capacity of DEN-TAT-PFC

**Figure 5:**
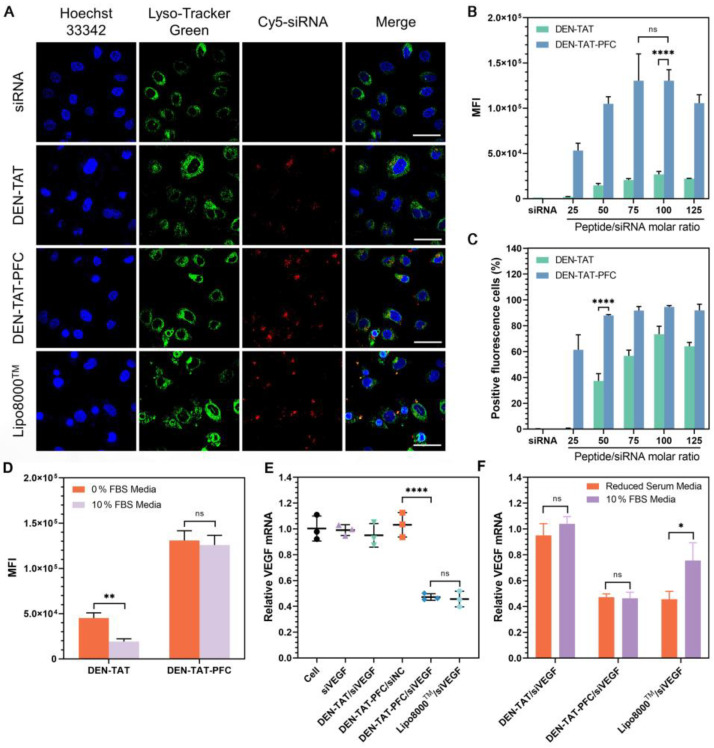
siRNA delivery efficiency of DEN-TAT-PFC: (**A**) Confocal microscopy images of the intracellular localization of DEN-TAT/siRNA and DEN-TAT-PFC/siRNA formed with siRNA labeled with Cy5 with a molar ratio of 100 in HepG2 cells. Hoechst 33342 dye (blue) was used to stain the cell nuclei, while LysoTracker Green was used to label endo(lyso)somes. The scale bar is 10 μm. Flow cytometry was used to determine the mean fluorescence intensity (MFI) of cells (**B**) and percentage of cells having Cy5 labeled siRNA (**C**) following incubation with DEN-TAT/siRNA and DEN-TAT-PFC/siRNA complexes at various molar ratios. (**D**) The impact of 10% FBS on the cellular uptake efficiency mediated by DEN-TAT and DEN-TAT-PFC. (**E**) Cells treated with different siRNA formulations had varying levels of VEGF mRNA. (**F**) The effect of serum (10% FBS) on the gene silencing efficiency of DEN-TAT and DEN-TAT-PFC. The mRNA levels were measured by real-time PCR. Data are expressed as mean ± SD (n = 3), ns represents insignificant, * *p* < 0.05, ** *p* < 0.01, **** *p* < 0.0001.

CLSM (Figure 5A) and flow cytometry (Figure 5B,C) were utilized to analyze the intracellular localization and cellular uptake of vector/siRNA complexes loaded with Cy5-labeled siRNA. The outcomes showed that both DEN-TAT and DEN-TAT-PFC were involved in the effective uptake of siRNA into cells. However, DEN-TAT-PFC/siRNA complexes accumulated into HepG2 cells more efficiently than DEN-TAT/siRNA complexes at all tested molar ratios (Figure 5A–C). Meanwhile, this phenomenon was also observed in other cells, such as 4T1 cells (Figure S4) and B16 cells (Figure S5). It was hypothesized that the fluorinated peptide exhibited good polar and non-polar phase separation properties [26], making them easily traverse the lipid bilayers of cells.

Vector/siRNA complexes are easily trapped into endosomes following cellular uptake via endocytosis. LysoTracker Green was used to assess the endosome escape capacity. In the colocalization image of DEN-TAT-PFC, there were several diffused cytoplasmic red spots that were barely observed in the cells treated with DEN-TAT, suggesting that fluorination can promote cell uptake and endosome escape ability (Figure 5A). Next, it displayed that the addition of FBS did not significantly interfere with the cell uptake efficacy of DEN-TAT-PFC. Conversely, it led to an apparent decrease in the uptake of DEN-TAT (Figure 5D).

Subsequently, the gene knockdown efficiency of different vectors was carried out using siRNA-targeting VEGF (siVEGF) with measurement of mRNA levels by quantitative RT-PCR. Successful knockdown of VEGF mRNA (approximately 53% reduction), where the siRNA delivery was mediated by DEN-TAT-PFC, whereas no gene knockdown happened for DEN-TAT at the same molar ratio (Figure 5E). More importantly, transfection assays were also compared in the presence of 10% FBS. And the results showed that the additional serum reduced the transfection efficiency of Lipo8000TM. However, relative mRNA levels of the DEN-TAT-PFC group were stable in either the reduced serum medium or 10% FBS medium, which further demonstrated the good serum resistance ability of the fluorinated carrier DEN-TAT-PFC (Figure 5F). Based on these data, the in vitro anti-tumor effect was also evaluated. The cytotoxicity of SF was concentration-dependent (Figure S6). Additionally, when the siRNA amount and vector/siRNA molar ratios increased, the viability of cells treated with DEN-TAT-PFC/siVEGF complexes decreased (Figures S7 and S8), and it revealed a better anti-tumor effect than DEN-TAT/siVEGF complexes at the same concentration and molar ratio (Figure S9). Taken together, these data validated that the DEN-TAT-PFC was a favorable siRNA delivery vector.

### 3.4. In Vitro Antitumor Efficacy and Mechanism

**Figure 6:**
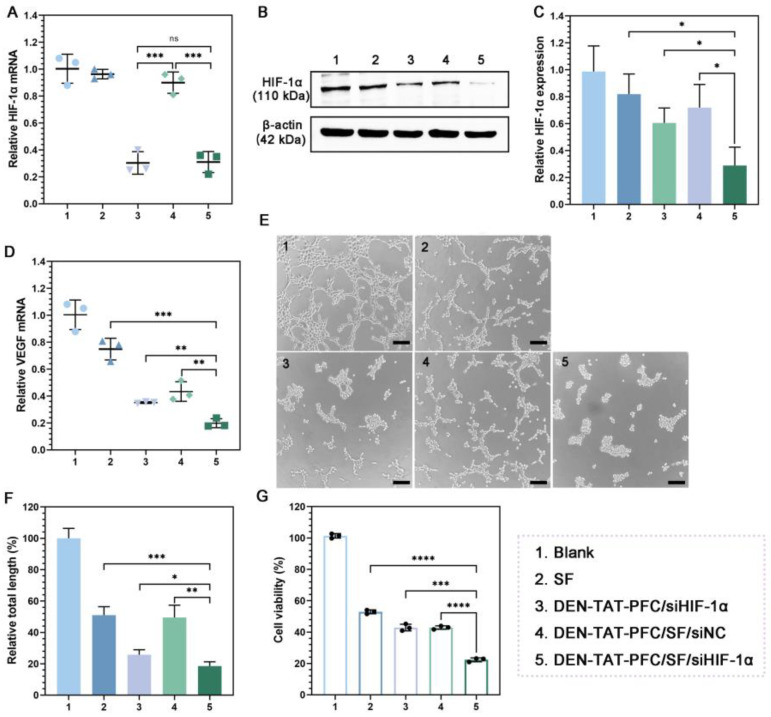
The HIF-1α and VEGF knockdown effect and antitumor efficacy: (**A**) Quantitative RT-PCR analysis of relative HIF-1α mRNA levels in HepG2 cells after treatment with SF, DEN-TAT-PFC/siHIF-1α, DEN-TAT-PFC/SF/siNC, and DEN-TAT-PFC/SF/siHIF-1α, respectively. (**B**) Western blot assay results for HIF-1α. The uncropped blots and molecular weight markers are shown in Supplementary Figure S12. (**C**) Semiquantitative analysis of HIF-1α levels. (**D**) Quantitative RT-PCR analysis of VEGF mRNA levels in HepG2 cells after multiple treatments. (**E**) The tubule formation of HUVEC on Matrix gel after incubation with various transfection mediums. Scale bar: 100 μm. (**F**) Semi-quantitative results of the tubule formation by relative tube length to the blank group calculated from Image J software. (**G**) The effects of different formulations on the viabilities of HepG2 cells within 48 h. The data are expressed as mean ± SD (*n* = 3). * *p* < 0.05, ** *p* < 0.01, *** *p* < 0.001, **** *p* < 0.0001.

Since the single DEN-TAT-PFC/siVEGF could not achieve satisfactory anti-tumor cell proliferation, siHIF-1α and SF co-delivered by DEN-TAT-PFC were further prepared. The mRNA level and protein expression of HIF-1α were analyzed by quantitative RT-PCR and WB assays. Data in Figure 6A indicated that HIF-1α mRNA levels decreased by approximately 70% for HepG2 cells transfected with DEN-TAT-PFC/siHIF-1α and DEN-TAT-PFC/SF/siHIF-1α. However, the SF alone or DEN-TAT-PFC/SF/siNC could not significantly downregulate the HIF-1α mRNA levels. Regarding HIF-1α protein levels, the tendency was somewhat different. As shown in the WB images and densitometric analysis (Figure 6B,C), the expression of HIF-1α was reduced by about 20% in cells treated with SF and DEN-TAT-PFC/SF/siNC. The reason was that SF was associated with the inhibition of HIF-1α protein synthesis rather than the promotion of HIF-1α protein degradation or the reduction of HIF-1α mRNA28. Additionally, DEN-TAT-PFC/SF/siHIF-1α exhibited an excellent HIF-1α protein silencing effect, which was superior to DEN-TAT-PFC/siHIF-1α and DEN-TAT-PFC/SF/siNC, suggesting a synergistic effect of SF and siHIF-1α.

Afterward, since the HIF-1α is a crucial transcription factor to inhibit angiogenesis, the influence of DEN-TAT-PFC/SF/siHIF-1α on the downstream VEGF expression was assessed. The VEGF mRNA levels were reduced after all the treatments (Figure 6D), especially in the DEN-TAT-PFC/SF/siHIF-1α group, where only about 20% of VEGF mRNA was detected. VEGF mRNA expression decreased by around 56% in the DEN-TAT-PFC/SF/siNC group, which was significantly lower than that in the SF alone group (about 25% reduction), indicating that the DEN-TAT-PFC vector could deliver more of SF to the tumor site for therapeutic effect. Therefore, both SF and siHIF-1α mediated by DEN-TAT-PFC could downregulate the VEGF, and the combination group could achieve the best VEGF mRNA silencing efficiency.

Next, the tube formation of HUVEC was examined with a conditioned medium of tumor cells after various treatments to estimate its angiogenic properties (Figure 6E,F). In the blank group, HUVEC formed an extensive tubular vascular network, a hallmark of angiogenesis. In contrast, the DEN-TAT-PFC/SF/siHIF-1α-treated group has few microvascular networks and shortest HUVEC alignments, indicating efficient anti-angiogenesis.

Inspired by these encouraging results, the anti-tumor activity of this co-loaded system was validated in vitro (Figure 6G). DEN-TAT-PFC/SF/siHIF-1α showed dramatic tumor-killing activity, compared with SF alone, DEN-TAT-PFC/SF/siNC, or DEN-TAT-PFC/siHIF-1α, which further revealed that the successful transfection of siHIF-1α, and in combination with SF, significantly increased the proliferation inhibition of HepG2 cells. Similar results were also obtained on two other human cancer cells (HeLa and A375 cells) (Figure S10). In addition, the co-delivery system showed much weaker cytotoxicity in the LO2 cells than in the HepG2 cells. This might be attributed to the specificity of SF to liver cancer cells; HIF-1α is not highly expressed in healthy human cells and releases less drug (weaker GSH-responsive) in normal cells than in cancer cells (Figure S11).

## 4. Conclusions

In conclusion, a well-designed and well-defined fluorinated cell-penetrating peptide DEN-TAT-PFC was synthesized to co-deliver siHIF-1α and sorafenib. Compared to non-fluorinated peptides, DEN-TAT-PFC exhibited an enhanced siRNA binding ability, GSH-responsive release, facilitated cellular uptake, endosome escape, serum-resistance ability, and oxygen-carrying capacity. Meanwhile, the biocompatible DEN-TAT-PFC was suitable for co-loading siHIF-1α and sorafenib to assemble cationic nanoscale complexes, which could effectively knockdown HIF-1α both at mRNA and protein levels and further suppress VEGF expression, which could realize TME reconstruction with both vascular normalization and hypoxia-relieving. Consequently, DEN-TAT-PFC/SF/siHIF-1α exhibited favorable anti-tumor cell proliferation and anti-angiogenesis via the synergetic effect. Altogether, DEN-TAT-PFC is a promising carrier for siRNA and fluorine-containing drugs for tumor treatment with targeting hypoxia.

## Data Availability

Data are contained within he article and supplementary materials.

## Supplementary Materials

The following supporting information can be downloaded at: https://www.mdpi.com/article/10.3390/pharmaceutics15122789/s1, Figure S1. The preparative HPLC chromatogram of its purification. Figure S2: Zeta potential of DEN-TAT-PFC/SF/siHIF-1α; Figure S3. (A) Live dead staining of HepG2 cells treated with DEN-TAT and DEN-TAT-PFC at concentrations of 8.75 μM. Scale bar: 100 μm. (B) Images after centrifugation of peptides incubated with 2% blood cell solution at different concentrations. Figure S4. Flow cytometry was used to determine (A) the percentage of cells carrying Cy5 labeled siRNA and (B) the mean fluorescence intensity (MFI) of 4T1 cells after incubation with DEN-TAT/siRNA complexes and DEN-TAT-PFC/siRNA complexes with varying molar ratios. Figure S5. Flow cytometry was used to determine (A) the percentage of cells carrying Cy5 labeled siRNA and (B) the mean fluorescence intensity (MFI) of B16 cells after incubation with DEN-TAT/siRNA complexes and DEN-TAT-PFC/siRNA complexes with varying molar ratios. Figure S6. The cell viability of HepG2 cells treated with different concentrations of sorafenib (1~50 μM). Figure S7. The cell viability of HepG2 cells treated with DEN-TAT-PFC/siVEGF formed at molar ratio 100 and different siRNA concentrations. Figure S8. The cell viability of HepG2 cells treated with DEN-TAT-PFC/siVEGF formed with 200 nM siVEGF at different molar ratios. Figure S9. The effects of different formulations on the viability of HepG2 cells. Figure S10. The cell viability of HeLa (A) and A375 cells (B) treated with different concentrations of sorafenib (1~50 μM). The effects of different formulations on the viabilities of HeLa cells (C) and A375 cells (D) within 48 h. A total of 15 μM sorafenib was used in HeLa cells, and 20 μM sorafenib was used in A375 cells. Figure S11. The cell viability of LO2 cells treated with SF, siHIF-1α, and DEN-TAT-PFC/SF/siHIF-1α; Figure S12. The raw data for Figure 6B (Western blot assay results for HIF-1α) in form of the uncropped blots and with molecular weight markers; Table S1. Mobile phase gradient elution program; Table S2. Specific primer sequences used in Quantitative Real-time PCR.

## References

[1] Shen J., Chen G.B., Zhao L.H., Huang G.Y., Liu H., Liu B.L., Miao Y.Q., Li Y.H. (2023). Recent Advances in Nanoplatform Construction Strategy for Alleviating Tumor Hypoxia. Adv. Healthc. Mater..

[2] Xu Q.Q., Lan X.Y., Lin H.M., Xi Q.Y., Wang M.C., Quan X.L., Yao G.Y., Yu Z.Q., Wang Y.X., Yu M. (2023). Tumor microenvironment-regulating nanomedicine design to fight multi-drug resistant tumors. Wiley Interdiscip. Rev. Nanomed. Nanobiotechnol..

[3] Bejarano L., Jordao M.J.C., Joyce J.A. (2021). Therapeutic Targeting of the Tumor Microenvironment. Cancer Discov..

[4] Peng S.J., Xiao F.F., Chen M.W., Gao H.L. (2022). Tumor-Microenvironment-Responsive Nanomedicine for Enhanced Cancer Immunotherapy. Adv. Sci..

[5] Carmeliet P. (2005). VEGF as a key mediator of angiogenesis in cancer. Oncology.

[6] Ribatti D., Annese T., Ruggieri S., Tamma R., Crivellato E. (2019). Limitations of Anti-Angiogenic Treatment of Tumors. Transl. Oncol..

[7] Liang Y.J., Zheng T.S., Song R.P., Wang J.B., Yin D.L., Wang L.L., Liu H.T., Tian L.T., Fang X., Meng X.Z. (2013). Hypoxia-Mediated Sorafenib Resistance Can Be Overcome by EF24 Through Von Hippel-Lindau Tumor Suppressor-Dependent HIF-1 alpha Inhibition in Hepatocellular Carcinoma. Hepatology.

[8] Mendez-Blanco C., Fondevila F., Garcia-Palomo A., Gonzalez-Gallego J., Mauriz J.L. (2018). Sorafenib resistance in hepatocarcinoma: Role of hypoxia-inducible factors. Exp. Mol. Med..

[9] Lee P., Chandel N.S., Simon M.C. (2020). Cellular adaptation to hypoxia through hypoxia inducible factors and beyond. Nat. Rev. Mol. Cell Bio..

[10] Feng W., Xue T., Huang S., Shi Q., Tang C., Cui G., Yang G., Gong H., Guo H. (2018). HIF-1alpha promotes the migration and invasion of hepatocellular carcinoma cells via the IL-8-NF-kappaB axis. Cell. Mol. Biol. Lett..

[11] Malekan M., Ebrahimzadeh M.A., Sheida F. (2021). The role of Hypoxia-Inducible Factor-1alpha and its signaling in melanoma. Biomed. Pharmacother..

[12] Mahdi A., Darvishi B., Majidzadeh-A K., Salehi M., Farahmand L. (2019). Challenges facing antiangiogenesis therapy: The significant role of hypoxia-inducible factor and MET in development of resistance to anti-vascular endothelial growth factor-targeted therapies. J. Cell Physiol..

[13] Chen X., Jin R., Jiang Q., Bi Q., He T., Song X., Barz M., Ai H., Shuai X., Nie Y. (2021). Delivery of siHIF-1alpha to Reconstruct Tumor Normoxic Microenvironment for Effective Chemotherapeutic and Photodynamic Anticancer Treatments. Small.

[14] Kang Z., Meng Q., Liu K. (2019). Peptide-based gene delivery vectors. J. Mater. Chem. B.

[15] Wan Y., Dai W., Nevagi R.J., Toth I., Moyle P.M. (2017). Multifunctional peptide-lipid nanocomplexes for efficient targeted delivery of DNA and siRNA into breast cancer cells. Acta Biomater..

[16] Cai X.J., Jin R.R., Wang J.L., Yue D., Jiang Q., Wu Y., Gu Z.W. (2016). Bioreducible fluorinated peptide dendrimers capable of circumventing various physiological barriers for highly efficient and safe gene delivery. ACS Appl. Mater. Interfaces.

[17] Hadianamrei R., Zhao X.B. (2022). Current state of the art in peptide-based gene delivery. J. Control. Release.

[18] Zhou L., Emenuga M., Kumar S., Lamantia Z., Figueiredo M., Emrick T. (2022). Designing Synthetic Polymers for Nucleic Acid Complexation and Delivery: From Polyplexes to Micelleplexes to Triggered Degradation. Biomacromolecules.

[19] Gorzkiewicz M., Konopka M., Janaszewska A., Tarasenko I.I., Sheveleva N.N., Gajek A., Neelov I.M., Klajnert-Maculewicz B. (2020). Application of new lysine-based peptide dendrimers D3K2 and D3G2 for gene delivery: Specific cytotoxicity to cancer cells and transfection in vitro. Bioorg. Chem..

[20] Han H., Xing J., Chen W., Jia J., Li Q. (2023). Fluorinated polyamidoamine dendrimer-mediated miR-23b delivery for the treatment of experimental rheumatoid arthritis in rats. Nat. Commun..

[21] Shi Z., Yang Y., Guo Z., Feng S., Wan Y. (2023). A cathepsin B/GSH dual-responsive fluorinated peptide for effective siRNA delivery to cancer cells. Bioorg. Chem..

[22] Wan Y., Yang Y., Wu M., Feng S. (2022). Fluorinated vectors for gene delivery. Expert Opin. Drug Deliv..

[23] Wu P.K., Luo X.P., Wu H., Zhang Q.Y., Dai Y.X., Sun M.J. (2020). Efficient and targeted chemo-gene delivery with self-assembled fluoro-nanoparticles for liver fibrosis therapy and recurrence. Biomaterials.

[24] Zhang T.B., Zhang Q., Tian J.H., Xing J.F., Guo W.S., Liang X.J. (2018). Perfluorocarbon-based nanomedicine: Emerging strategy for diagnosis and treatment of diseases. Mrs Commun..

[25] Gao M., Liang C., Song X.J., Chen Q., Jin Q.T., Wang C., Liu Z. (2017). Erythrocyte-Membrane-Enveloped Perfluorocarbon as Nanoscale Artificial Red Blood Cells to Relieve Tumor Hypoxia and Enhance Cancer Radiotherapy. Adv. Mater..

[26] Xiong S.D., Li L., Jiang J., Tong L.P., Wu S.L., Xu Z.S., Chu P.K. (2010). Cationic fluorine-containing amphiphilic graft copolymers as DNA carriers. Biomaterials.

