# Supplementary material for: Fluorinated Cell-Penetrating Peptide for Co-Delivering siHIF-1α and Sorafenib to Enhance In Vitro Anti-Tumor Efficacy
Source: Pharmaceutics. 2023 Dec 16;15(12):2789. doi: 10.3390/pharmaceutics15122789 (PMC10747150; doi:10.3390/pharmaceutics15122789)
Supplement: Supplementary file 1 [file pharmaceutics-15-02789-s001.zip › pharmaceutics-2771913-supplementary.pdf]

Supporting Information for

Fluorinated peptide for co-delivering siHIF-1α and sorafenib to enhance in vitro anti-tumor efficacy

Yu Wan, Yuhan Yang, Qiuyue Lai, Wangxia Wang, Mingyu Wu, Shun Feng*

Sichuan Engineering Research Center for Biomimetic Synthesis of Natural Drugs, School of Life Science and Engineering, Southwest Jiaotong University, Chengdu 610031, China.

Supplementary Figures

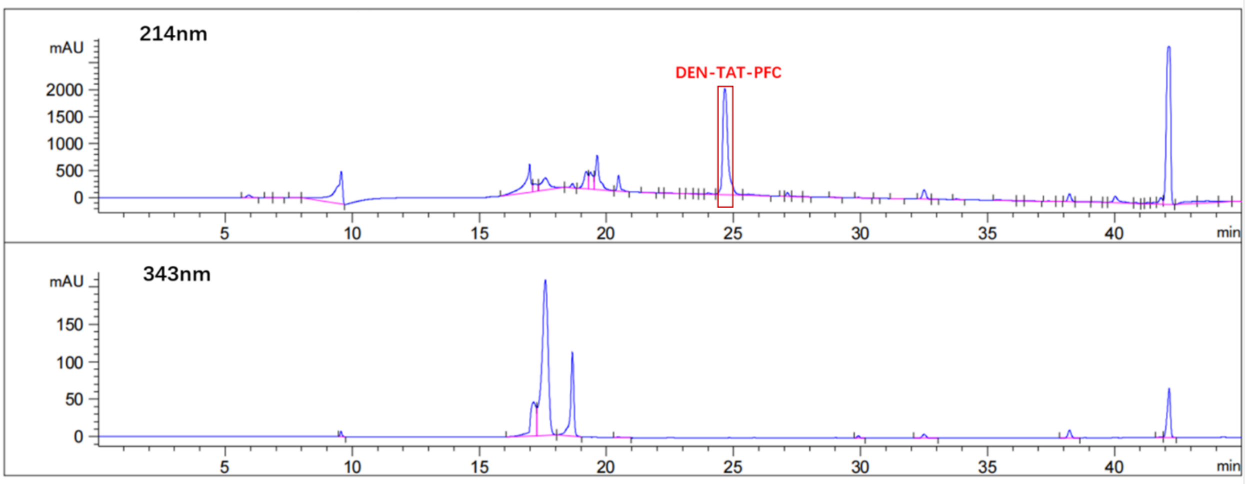

Figure S1. The preparative HPLC chromatogram of its purification. The red circle labeled peak was collected as the product of DEN-TAT-PFC. The peptides DEN-TAT, DEN-TAT-PFC, and PFC were detected at 214 nm and the byproduct pyridine-2-thione was detected at 343 nm.

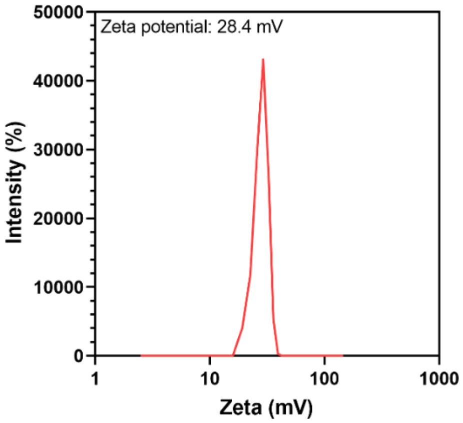

Figure S2. Zeta potential of DEN-TAT-PFC/SF/siHIF-1α.

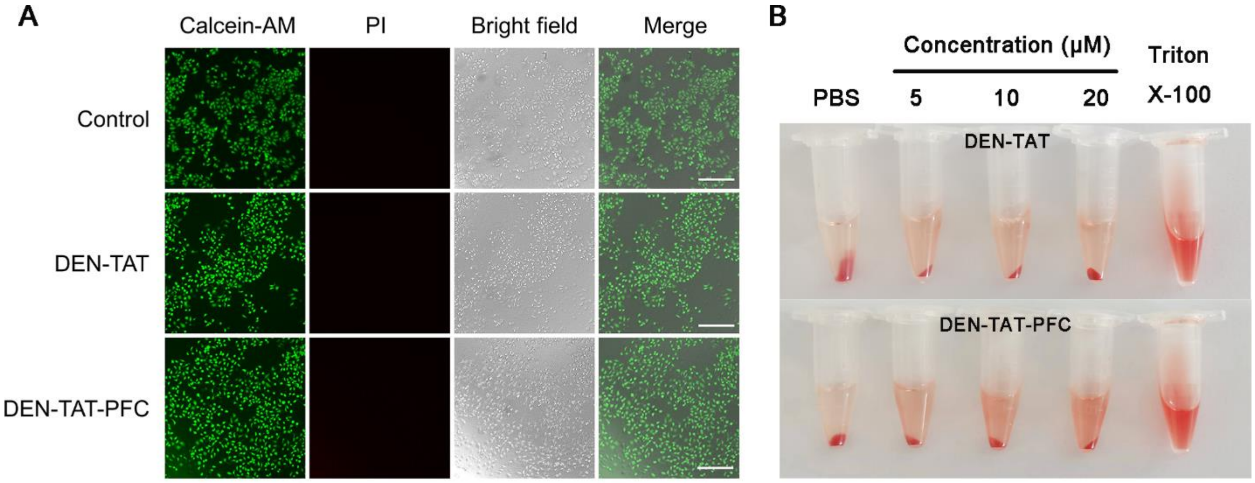

Figure S3. (A) Live dead staining of HepG2 cells treated with DEN-TAT and DEN-TAT-PFC at concentra-tions of 8.75 μM. Scale bar: 100 μm. (B) Images after centrifugation of peptides incubated with 2% blood cell solution at different concentrations.

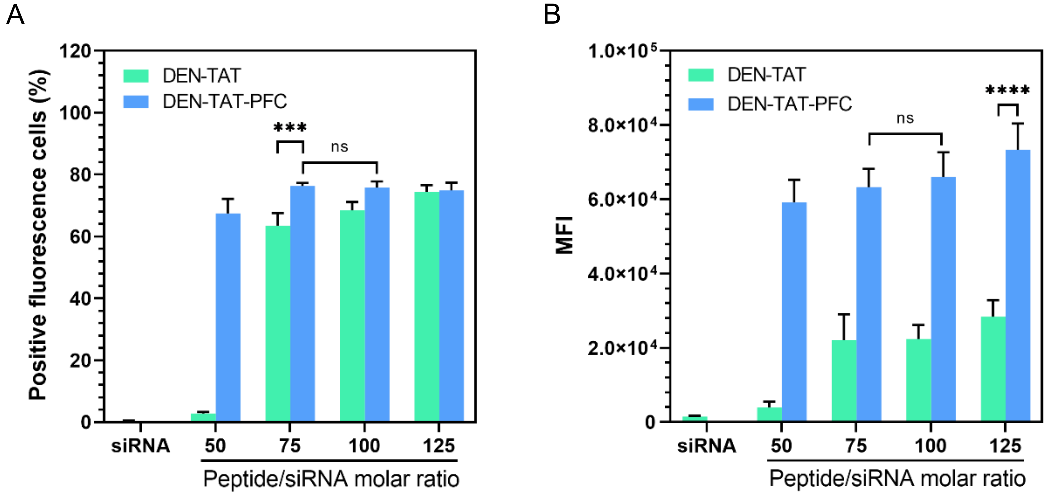

Figure S4. (A) The percentage of cells containing Cy5 labeled siRNA and (B) mean fluorescence intensity (MFI) of 4T1 cells after incubation with DEN-TAT/siRNA and DEN-TAT-PFC/siRNA complexes at different molar ratios (50, 75, 100 and 125), quantified by flow cytometry.

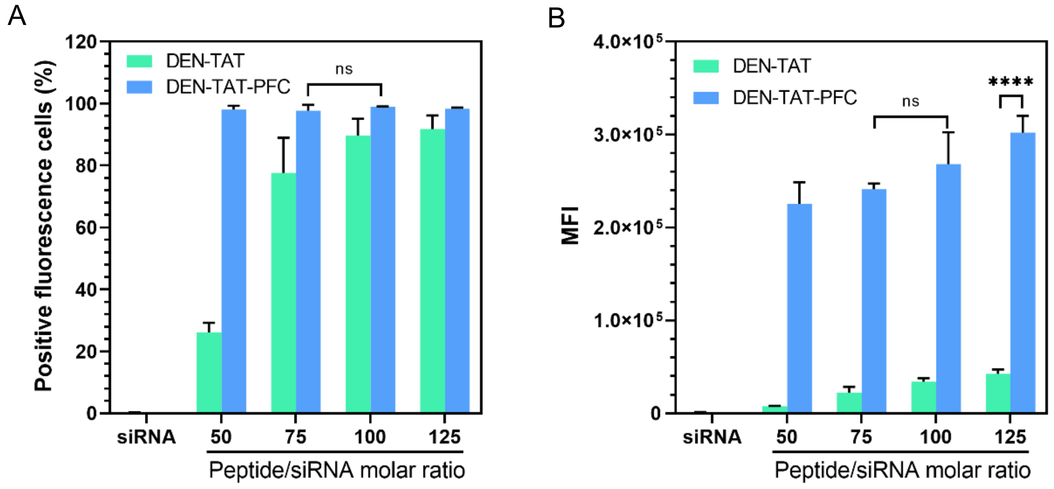

Figure S5. (A) The percentage of cells containing Cy5 labeled siRNA and (B) mean fluorescence intensity (MFI) of B16 cells after incubation with DEN-TAT/siRNA and DEN-TAT-PFC/siRNA complexes at different molar ratios (50, 75, 100 and 125), quantified by flow cytometry.

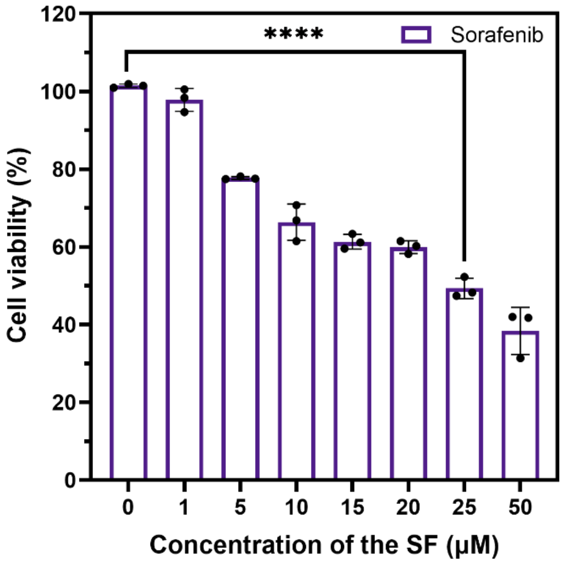

Figure S6. The cell viability of HepG2 cells treated with different concentration of sorafenib (1~50 μM).

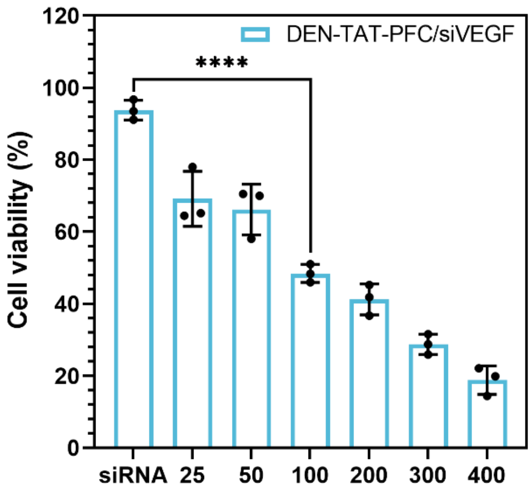

Figure S7. The cell viability of HepG2 cells treated with DEN-TAT-PFC/siVEGF formed at molar ratio 100 and different siRNA concentrations.

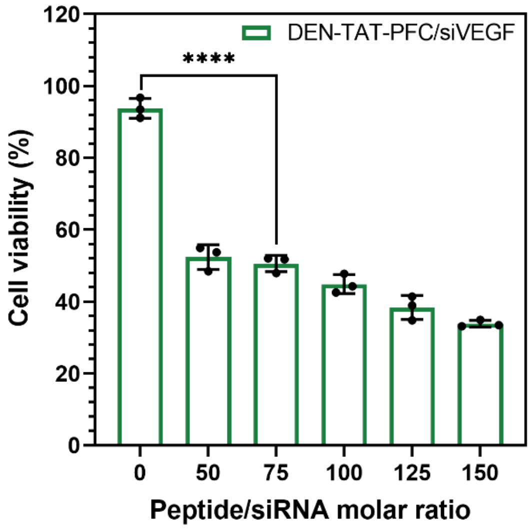

Figure S8. The cell viability of HepG2 cells treated with DEN-TAT-PFC/siVEGF formed with 200 nM siVEGF at different molar ratios.

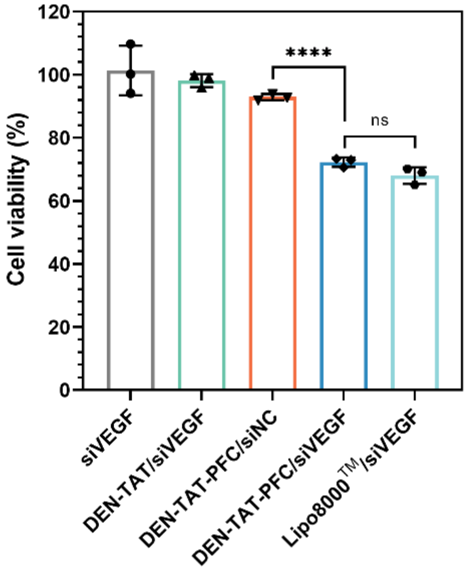

Figure S9. The effects of different formulations on the viability of HepG2 cells.

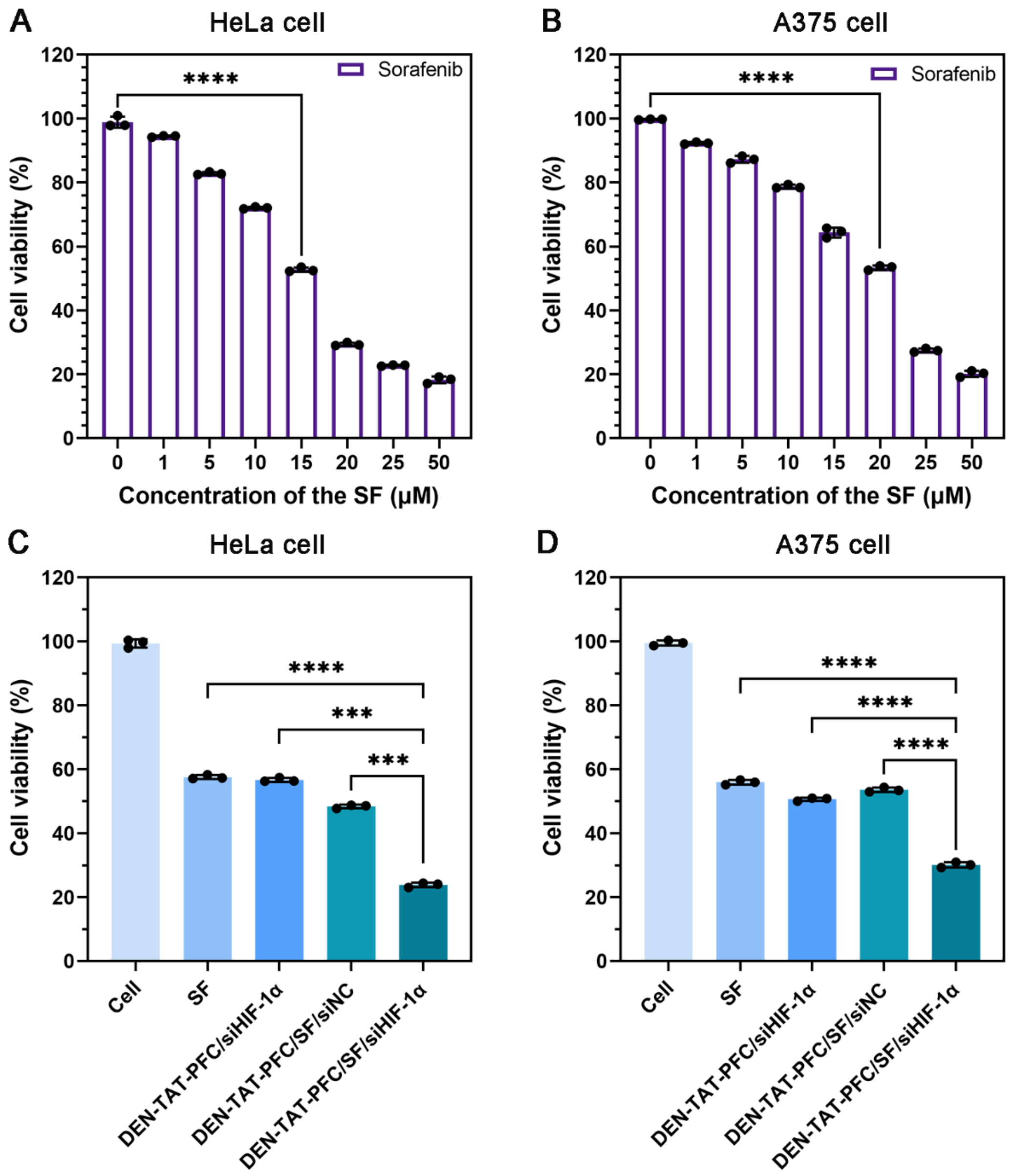

Figure S10. The cell viability of HeLa (A) and A375 cells (B) treated with different concentration of sorafenib (1~50 μM). The effects of different formulations on the viabilities of HeLa cells (C) and A375 cells (D) within 48 h. 15 μM sorafenib was used in HeLa cells, and 20 μM sorafenib was used in A375 cells. The data are expressed as mean ± SD (n = 3). ***$p < 0.001$, ****$p < 0.0001$.

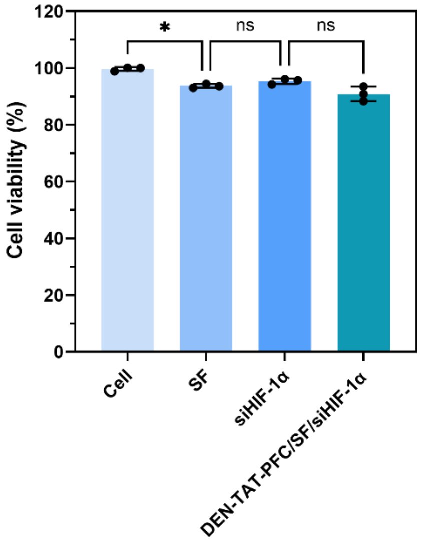

Figure S11. The cell viability of LO2 cells treated with SF, siHIF-1α and DEN-TAT-PFC/SF/siHIF-1α. The data are expressed as mean ± SD (n = 3). ns represents no significant difference, *p < 0.05.

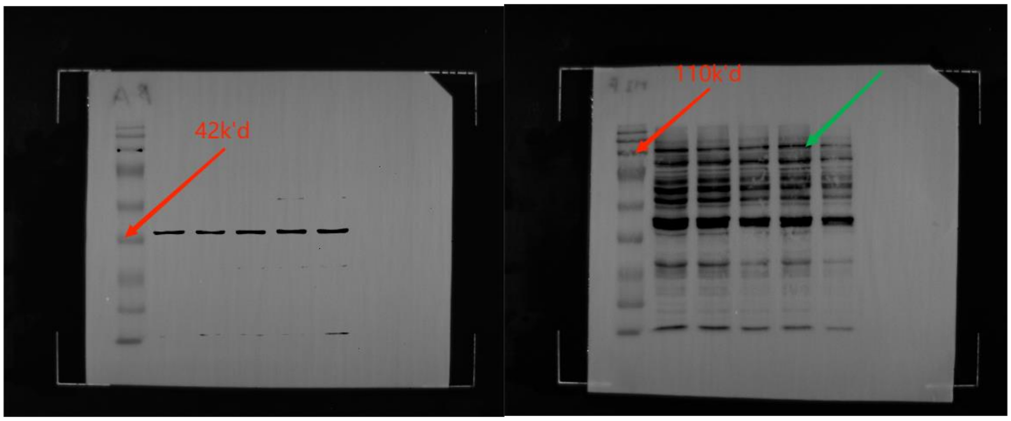

Figure S12. The raw data for Figure 6B (Western blot assay results for HIF-1α) in form of the uncropped blots and with molecular weight markers.

Supplementary Tables

Table S1. Mobile phase gradient elution program

Time (min)	Flow Rate (mL/min)	Water + 0.1% TFA (%)	Acetonitrile + 0.1% TFA (%)
0	2.0	100.0	0.0
5	2.0	90.0	10.0
30	2.0	20.0	80.0
35	2.0	0.0	100.0
42	2.0	0.0	100.0
45	2.0	100.0	0.0

Table S2. Specific primer sequences used in Quantitative Real-time PCR

**Gene**	**Forward primer**	**Reverse primer**
VEGF	5'- AGG AGG GCA GAA TCA TCA CG -3'	5'- GAT CCG CAT AAT CTG CAT GGT -3'
HIF-1α	5'- TCA CCA CAG GAC AGT ACA GGA TGC -3'	5'- CCA GCA AAG TTA AAG CAT CAG GTT CC -3'
GAPDH	5'- CCA AGG TCA ACC ATG ACA AC -3'	5'- TCC ACA GTC TTC TGA GTG GC -3'
β-actin	5'- GTG GGG CGC CCC AGG CAC CAG GGC -3'	5'- CTC CTT AAT GTC ACG CAC GAT TTC -3'
